# Rejected Online Feedback From a Swiss Physician Rating Website Between 2008 and 2017: Analysis of 2352 Ratings

**DOI:** 10.2196/18374

**Published:** 2020-08-03

**Authors:** Stuart McLennan

**Affiliations:** 1 Institute of History and Ethics in Medicine Technical University of Munich Munich Germany; 2 Institute for Biomedical Ethics University of Basel Basel Switzerland

**Keywords:** physician rating websites, patient satisfaction, participatory medicine, patient feedback

## Abstract

**Background:**

Previous research internationally has only analyzed publicly available feedback on physician rating websites (PRWs). However, it appears that many PRWs are not publishing all the feedback they receive. Analysis of this rejected feedback could provide a better understanding of the types of feedback that are currently not published and whether this is appropriate.

**Objective:**

The aim of this study was to examine (1) the number of patient feedback rejected from the Swiss PRW Medicosearch, (2) the evaluation tendencies of the rejected patient feedback, and (3) the types of issues raised in the rejected narrative comments.

**Methods:**

The Swiss PRW Medicosearch provided all the feedback that had been rejected between September 16, 2008, and September 22, 2017. The feedback were analyzed and classified according to a theoretical categorization framework of physician-, staff-, and practice-related issues.

**Results:**

Between September 16, 2008, and September 22, 2017, Medicosearch rejected a total of 2352 patient feedback. The majority of feedback rejected (1754/2352, 74.6%) had narrative comments in the German language. However, 11.9% (279/2352) of the rejected feedback only provided a quantitative rating with no narrative comment. Overall, 25% (588/2352) of the rejected feedback were positive, 18.7% (440/2352) were neutral, and 56% (1316/2352) were negative. The average rating of the rejected feedback was 2.8 (SD 1.4). In total, 44 subcategories addressing the physician (n=20), staff (n=9), and practice (n=15) were identified. In total, 3804 distinct issues were identified within the 44 subcategories of the categorization framework; 75% (2854/3804) of the issues were related to the physician, 6.4% (242/3804) were related to the staff, and 18.6% (708/3804) were related to the practice. Frequently mentioned issues identified from the rejected feedback included (1) satisfaction with treatment (533/1903, 28%); (2) the overall assessment of the physician (392/1903, 20.6%); (3) recommending the physician (345/1903, 18.1%); (4) the physician’s communication (261/1903, 13.7%); (5) the physician’s caring attitude (220/1903, 11.6%); and (6) the physician’s friendliness (203/1903, 10.6%).

**Conclusions:**

It is unclear why the majority of the feedback were rejected. This is problematic and raises concerns that online patient feedback are being inappropriately manipulated. If online patient feedback is going to be collected, there needs to be clear policies and practices about how this is handled. It cannot be left to the whims of PRWs, who may have financial incentives to suppress negative feedback, to decide which feedback is or is not published online. Further research is needed to examine how many PRWs are using criteria for determining which feedback is published or not, what those criteria are, and what measures PRWs are using to address the manipulation of online patient feedback.

## Introduction

There remains relevant unwarranted variation in health care systems and deficiencies regarding all key aspects of health care [1]. Members of the public, however, have traditionally had few ways of knowing who the “good” health care organizations and professionals are [2]. As part of a wider move toward transparency, public reporting activities have been developed in a number of countries with the aim of providing information about health care organizations or professionals to the public to correct this asymmetry of information in order to inform patient decision-making and drive quality improvement [3-6].

One type of public reporting activity that has been developed in recent decades is physician rating websites (PRWs) [7-10]. PRWs represent a “bottom-up” approach to public reporting, allowing users to post ratings and comments regarding their physician as a source of information for others [11-14]. Although patients have always been able to share their opinions about their physicians with others, the ability to share these opinions via the internet and social media now means that these opinions have the potential to reach a far wider audience. With a growing number of patients utilizing the internet in relation to health care [15], it is expected that PRWs will play an increasingly important role.

A recent systematic search of PRWs internationally analyzed 143 different websites from 12 countries [16] and found that the majority (76.9%) of websites provided options to give feedback both on a predefined quantitative rating scale and as narrative comments. Previous research internationally has often focused on analyzing the ratings and comments publicly available on PRWs. This research has reported that many PRWs have incomplete lists of physicians, a low number of physicians rated, and a low number of ratings per physician that are overwhelmingly positive, which has raised concerns about the representativeness, validity, and usefulness of information on PRWs [14,17]. Furthermore, the medical profession has often expressed concerns that feedback on PRWs will be manipulated for “doctor bashing” or defamation [10].

The first PRWs in Switzerland were established in 2008, at the same time as many international PRWs. However, in comparison with other countries that have established PRWs, there has been limited research conducted on PRWs in Switzerland. This author recently conducted a study involving a random stratified sample of 966 physicians generated from the regions of Zürich and Geneva [18,19]. Selected physicians were searched on a total of four websites (OkDoc, Medicosearch, DocApp, and Google) between November 2017 and July 2018, and it was recorded whether the physician could be found. Moreover, the physician’s rating, the number of ratings and narrative comments, and the text of narrative comments were recorded. As far as the author is aware, this was the first inclusion of Google in a study examining physician ratings internationally.

With regard to the frequency of quantitative ratings and narrative comments on Swiss PRWs, similar issues as those identified in the international literature were found. Many of the selected physicians could not be identified (the proportion of physicians who could be identified ranged from 42.4% on OkDoc to 87.3% on DocApp), few of the identifiable physicians had been rated quantitatively (4.5% on DocApp to 49.8% on Google) or received a narrative comment (4.5% on DocApp to 31.2% on Google) at least once, rated physicians had, on average, a low number of quantitative ratings (1.47 ratings on OkDoc to 3.74 rating on Google) and narrative comments (1.23 comments on OkDoc to 3.03 comments on Google), and all three websites that allowed quantitative ratings had very positive average ratings on a 5-star rating scale (DocApp, 4.71; Medicosearch, 4.69; and Google, 4.41) [18].

With regard to the contents of narrative comments, it was found that the selected physicians had a total of 849 comments [19]. Narrative comments were analyzed and classified according to a theoretical categorization framework previously developed by Emmert et al [10]. In total, 43 subcategories addressing the physician, staff, and practice were identified. None of the PRWs’ comments covered all 43 subcategories of the categorization framework; comments on Google covered 86% of the subcategories, those on Medicosearch covered 72%, those on DocApp covered 60%, and those on OkDoc covered 56%. In total, 2441 distinct issues were identified within the 43 subcategories of the categorization framework; 83.65% of the issues were related to the physician, 6.63% were related to the staff, and 9.70% were related to the practice. Overall, 95% of the subcategories of the categorization framework and 81.60% of the distinct issues identified were concerning aspects of performance (interpersonal skills of the physician and staff, infrastructure, and organization and management of the practice) considered assessable by patients [19]. Furthermore, this research raised concerns that user feedback is being suppressed by Swiss PRWs [18,19], which risks undermining the overall aim of PRWs of providing a reliable source of unbiased information regarding patients’ experiences and satisfaction with physicians.

As far as this author is aware, previous research internationally has only analyzed publicly available feedback on PRWs. However, as it appears that many PRWs are not publishing all the feedback they receive. Analysis of this rejected feedback could provide a better understanding of the types of feedback that are currently not published and whether this is appropriate. This study therefore aimed to examine (1) the number of patient feedback rejected from the Swiss PRW Medicosearch, (2) the evaluation tendencies of the rejected patient feedback, and (3) the types of issues raised in the rejected narrative comments. Gaining a better understanding of feedback rejected by PRWs may help to identify issues in the way PRWs are currently determining which feedback are and are not to be publicly published.

## Methods

### Sample

Switzerland is a Central European country with a population of about 8.4 million people and 4 official languages (German, French, Italian, and Romansh). The Swiss health care system is highly complex and decentralized, and all Swiss residents are required to purchase basic mandatory health insurance that is offered by competing nonprofit insurers. Mandatory health insurance covers most general practitioner and specialist services, and people not enrolled in managed care plans generally have free choice of professionals [18]. The first PRWs in Switzerland, OkDoc and Medicosearch, were established in 2008. A systematic web-based search conducted in June 2016 identified that the websites DocApp and Google also allow users to view quantitative ratings and/or narrative comments about Swiss physicians in a structured manner without having to open an account or log onto the website [18,19]. It appears that other websites have also subsequently started to allow users to view quantitative ratings and/or narrative comments about Swiss physicians (eg, DeinDoktor and Doctena) [19]. Nevertheless, out of the dedicated Swiss PRWs, Medicosearch appears to be one of the best established and used [18,19]. Medicosearch allows users to search for physicians by location and specialty. Physician profiles provide general information about the physician (specialties, languages spoken, and contact details). In recent years, Medicosearch has shifted its business strategy toward online appointments, where physicians pay a fee and their booking systems are integrated with Medicosearch, allowing patients to book an appointment with a physician directly on Medicosearch. Users can also leave reviews of physicians, but Medicosearch requires both a quantitative rating (5-star rating scale) and a narrative comment in every patient feedback. Although Medicosearch allows negative comments, it informs the concerned physician before publishing it on the website, so that the physician can decide whether to activate the negative feedback function. If the physician refuses, the feedback function is deactivated, removing positive comments as well [19].

As part of a larger project examining Swiss PRWs, the author approached the CEO of Medicosearch, Beat Burger. Discussions confirmed that Medicosearch does not publish all the feedback they receive. The author enquired about the possibility of receiving these rejected feedback for analysis. Medicosearch agreed to provide the rejected feedback to the author in an anonymous form. On October 24, 2017, Medicosearch sent the author an excel file that included all the feedback that Medicosearch had rejected between September 16, 2008, and September 22, 2017. The details of the rated physicians were not included. Medicosearch did not provide any reasons for why the feedback were rejected. The following data were imported into a Statistical Package for the Social Sciences (SPSS version 26 for Windows, IBM Corporation) file: the date the feedback was created, the quantitative rating out of 5, and the narrative comments provided under “title” and “description.” In early 2019, the author inquired with Medicosearch whether it would be possible to receive the rejected feedback from September 23, 2017, to December 31, 2018. Medicosearch told the author on April 11, 2019, that owing to new data-protection rules, Medicosearch had deleted all rejected feedback, and therefore, it was not possible to provide an updated file.

### Data Analysis

Medicosearch uses a 5-star rating scale; a rating of 4 to 5 stars was considered a positive rating, 3 stars was considered a neutral rating, and 1 to 2 stars was considered a negative rating. The content of each narrative comment was analyzed and classified by the author according to a theoretical categorization framework of physician-, staff-, and practice-related issues. The categorization framework from Emmert et al was initially used [10], with modifications where necessary. This included removing categories that were not identified in the comments, adding categories that were identified but were not adequately covered by the previous framework, and separating categories (eg, friendliness and caring attitude) that were discussed in comments as distinct issues. Narrative comments were analyzed in their original language. Descriptive statistics included means and standard deviations for continuous variables and percentages for categorical variables. To analyze whether differences existed between German and French comments, chi-squared tests were used. All analyses were performed with the significance level α set to .05 and two-tailed tests, using SPSS version 26.

## Results

### Characteristics of Ratings

Between September 16, 2008, and September 22, 2017, Medicosearch rejected a total of 2352 patient feedback (Table 1).

The majority of feedback rejected (1754/2352, 74.6%) had narrative comments in German (Table 2). Other rejected feedback had narrative comments in French (275/2352, 11.7%), Italian (31/2352, 1.3%), English (12/2352, 0.5%), and Spanish (1/2352, 0.04%). However, 11.9% (279/2352) of the rejected feedback only provided a quantitative rating with no narrative comment.

Overall, 25% (588/2352) of the quantitative ratings were positive, 18.7% (440/2352) were neutral, and 56% (1316/2352) were negative (Table 3). Additionally, the average rating of the rejected feedback was 2.8 (SD 1.4).

**Table 1:** Distribution of rejected feedback according to year.

Distribution of comments (year)^a^ (N=2352), n (%)									
2008	2009	2010	2011	2012	2013	2014	2015	2016	2017
26 (1.1)	259 (11.0)	232 (9.9)	344 (14.6)	392 (16.7)	321 (13.6)	268 (11.4)	142 (6.0)	236 (10.0)	132 (5.6)

^a^From September 16, 2008, to September 22, 2017.

**Table 2:** Distribution of rejected feedback according to language.

Language (N=2352), n (%)					
German	French	Italian	English	Spanish	Missing
1754 (74.6)	275 (11.7)	31 (1.3)	12 (0.5)	1 (0.04)	279 (11.9)

**Table 3:** Quantitative rating evaluation results.

Measure		Language^a^							Total (N=2352), n (%)
		German (N=1754), n (%)	French (N=275), n (%)	Italian (N=31), n (%)	English (N=12), n (%)	Spanish (N=1), n (%)	Missing (N=279), n (%)		
**Evaluation**									
	Positive	399 (22.7)	81 (29.5)	4 (12.9)	4 (33.3)	1/1 (100)	99 (35.5)	588 (25.0)	
	Neutral	296 (16.9)	59 (21.5)	6 (19.4)	1 (8.3)	0 (0)	77 (26.3)	440 (18.7%)	
	Negative	1065 (60.4)	135 (49.1)	21 (67.7)	7 (58.3)	0 (0)	88 (30)	1316 (56%)	
	Missing	0 (0)	0 (0)	0 (0)	0 (0)	0 (0)	8 (2.9)	8 (0.3)	
Average rating (SD)		2.7 (1.3)	3.0 (1.4)	2.4 (1.3)	2.9 (1.5)	5.0	3.2 (1.4)	2.8 (1.4)	

Of the 2073 ratings that provided narrative comments, analysis found that a total of 170 comments were not feedback from a patient concerning the physician, staff, or practice (92 comments were not comprehensible, 29 comments were explicitly labelled as test ratings, 15 comments were about the PRW, 10 comments reported that the person had not visited the physician yet, 10 comments simply reported that the physician’s details were not up to date, eight comments were abusive, four comments were second-hand reports, two comments were asking for advice regarding their or their family member’s condition, and two comments were not concerning a visit to a physician). Consequently, this feedback was excluded from the categorization framework.

The 1903 included narrative comments had a mean length of 158 characters (SD 214), ranging from 1 to 2788 characters. There was a significant difference in the mean character length between positive comments (mean 88, SD 130) and negative comments (mean 193, SD 241) (t_1314_=−11, *P*<.001). There was no significant difference in the mean character length between German comments (mean 158, SD 205) and French comments (mean 154, SD 206) (t_1862_=0.2, *P*=.82).

### Categorization of Issues

Content analysis of the included 1903 narrative comments identified 44 subcategories addressing the physician (n=20), staff (n=9), and practice (n=15) (Textbox 1).

**Categorization framework.**

**Physician (n=20)**

Satisfaction with treatment; overall assessment; recommendation; communication; caring attitude; friendliness; competence; treatment cost/billing; being taken seriously; time spent with patient; trust; professionalism; cooperation with medical specialists; alternative medicine; telephone availability; privacy; health insurance differentiation; patient involvement; individualized service; child friendliness

**Staff (n=9)**

Friendliness; overall assessment; service/assistance; communication; professionalism; availability by telephone; time spent with patient; health insurance differentiation; trust

**Practice (n=15)**

Overall assessment; waiting time within the practice; atmosphere; organization; ability to get appointment; equipment; recommendation; consultation hours; location; waiting room entertainment; parking space; availability by telephone; privacy; barrier-free access; online appointment

In total, 3804 distinct issues were identified within the 44 subcategories of the categorization framework; 75% (2854/3804) of the issues were related to the physician, 6.4% (242/3804) were related to the staff, and 18.6% (708/3804) were related to the practice (Table 4). The most frequent issue mentioned in the rejected comments was satisfaction with treatment (533/1903, 28%); 73.2% (390/533) of these ratings were negative. Other frequently mentioned issues regarding the physician were as follows: 20.6% (392/1903) of comments provided an overall assessment of the physician (53.8% negative); 18.1% (345/1903) provided a recommendation regarding the physician (76.2% negative); 13.7% (261/1903) referred to the physician’s communication (77.8% negative); 11.6% (220/1903) referred to the physician’s caring attitude (75.9% negative); and 10.6% (203/1903) referred to the physician’s friendliness (73.9% negative). In relation to staff issues, the most frequently mentioned issue was regarding the staffs’ friendliness (109/1903; 5.7%); 43.1% of these ratings were negative. Concerning practice issues, the frequently mentioned issues were as follows: 15.5% (295/1903) of the comments provided an overall assessment (38.6% positive), 8.1% (155/1903) referred to the waiting time within the practice (58.7% negative), and 5% (96/1903) referred to the atmosphere of the practice (40.6% positive).

However, there were some significant differences between German and French comments in a number of subcategories (Multimedia Appendix 1). For instance, German comments referred significantly more often to the physician’s friendliness (χ^2^_1_=5.9, *P*=.01), being taken seriously by the physician (χ^2^_1_=8.5, *P*=.002), staff friendliness (χ^2^_1_=7.0, *P*=.005), waiting time in the practice (χ^2^_1_=6.3, *P*=.01), and practice atmosphere (χ^2^_1_=6.6, *P*=.007).

**Table 4:** Categorization of issues.

Issue		Total (N=1903), n (%)	Quantitative rating evaluation		
			Positive, n (%)	Neutral, n (%)	Negative, n (%)
**Physician**					
	Satisfaction with treatment	533 (28.0)	82 (15.4)	61 (11.4)	390 (73.2)
	Overall assessment	392 (20.6)	122 (31.0)	59 (15.1)	211 (53.8)
	Recommendation	345 (18.1)	35 (10.1)	47 (13.6)	263 (76.2)
	Communication	261 (13.7)	39 (14.9)	19 (7.3)	203 (77.8)
	Caring attitude	220 (11.6)	40 (18.2)	13 (5.9)	167 (75.9)
	Friendliness	203 (10.6)	35 (17.2)	18 (8.9)	150 (73.9)
	Treatment cost/billing	173 (9.1)	8 (4.6)	30 (17.3)	135 (78.0)
	Competence	170 (8.9)	43 (25.3)	13 (7.6)	114 (67.1)
	Being taken seriously	141 (7.4)	10 (7.1)	10 (7.1)	121 (85.8)
	Time spent with patient	136 (7.1)	17 (12.5)	18 (13.2)	101 (74.3)
	Trust	133 (6.9)	44 (33.1)	11 (8.3)	78 (58.6)
	Professionalism	97 (5.1)	11 (11.3)	6 (6.2)	80 (82.5)
	Cooperation with medical specialists	13 (0.7)	4 (30.8)	0	9 (69.2)
	Alternative medicine	8 (0.4)	6 (75.0)	0	2 (25.0)
	Telephone availability	8 (0.4)	1 (12.5)	3 (37.5)	4 (50.0)
	Privacy	7 (0.4)	0	2 (28.6)	5 (71.4)
	Health insurance differentiation	6 (0.3)	0	0	6 (100)
	Patient involvement	5 (0.3)	0	1 (20.0)	4 (80.0)
	Individualized service	2 (0.1)	0	0	2 (100)
	Child friendliness	1 (0.04)	0	0	1 (100)
**Staff**					
	Friendliness	109 (5.7)	39 (35.8)	23 (21.1)	47 (43.1)
	Overall assessment	60 (3.2)	19 (31.7)	18 (30.0)	23 (38.3)
	Service/assistance	31 (1.6)	11 (35.5)	3 (9.7)	17 (54.8)
	Communication	22 (1.2)	4 (18.2)	7 (31.8)	11 (50.0)
	Professionalism	10 (0.5)	2 (20.0)	2 (20.0)	6 (60.0)
	Availability by telephone	6 (0.3)	1 (16.7)	4 (66.7)	1 (16.7)
	Time spent with patient	2 (0.1)	1 (50)	0	1 (50.0)
	Health insurance differentiation	1 (0.04)	0	1 (100)	0
	Trust	1 (0.04)	0	0	1 (100)
**Practice**					
	Overall assessment	295 (15.5)	114 (38.6)	90 (30.5)	91 (30.8)
	Waiting time within practice	155 (8.1)	22 (14.2)	42 (27.1)	91 (58.7)
	Atmosphere	96 (5.0)	39 (40.6)	29 (30.2)	28 (29.2)
	Organization	37 (1.9)	7 (18.9)	11 (29.7)	19 (51.4)
	Ability to get appointment	36 (1.9)	4 (11.1)	11 (30.6)	21 (58.3)
	Equipment	31 (1.6)	8 (25.8)	10 (32.3)	13 (41.9)
	Recommendation	25 (1.3)	4 (16.0)	5 (20.0)	16 (64.0)
	Consultation hours	8 (0.4)	3 (37.5)	3 (37.5)	2 (25.0)
	Location	7 (0.4)	2 (28.6)	2 (28.6)	3 (42.9)
	Waiting room entertainment	5 (0.3)	4 (80.0)	1 (20.0)	0
	Parking space	4 (0.2)	4 (100)	0	0
	Availability by telephone	3 (0.2)	1 (33.3)	1 (33.3)	1 (33.3)
	Privacy	3 (0.2)	0	3 (100)	0
	Barrier-free access	2 (0.1)	0	1 (50.0)	1 (50.0)
	Online appointment	1 (0.04)	0	1 (100)	0

## Discussion

### Principal Findings

As far as this author is aware, this is the first study internationally to examine feedback that has been rejected from a PRW. The key findings of this study are as follows: (1) the Swiss PRW Medicosearch rejected a total of 2352 patient feedback between September 16, 2008, and September 22, 2017, (2) just over half of all the rejected feedback were negative, and (3) the most frequently mentioned issue in the rejected feedback was satisfaction with treatment. Medicosearch has shown a lot of transparency in providing this rejected feedback for analysis. It is, however, unclear why the majority of the feedback were rejected. This is problematic and raises concerns that online patient feedback are being inappropriately manipulated.

Medicosearch did not provide the reasons why it rejected the feedback, and as far as this author is aware, Medicosearch does not use formal criteria for determining which feedback should be published or rejected. Of the 2352 ratings rejected by Medicosearch, 170 comments were excluded from the categorization framework for various reasons. For example, the feedback were not comprehensible, were explicitly labelled as test ratings, were about the PRW, etc. These would also appear to be legitimate reasons for Medicosearch to reject the feedback. However, the appropriateness of rejecting the remaining 92.7% of feedback is less clear, particularly as they appear to be qualitatively the same as the published feedback for a sample of Swiss physicians recently analyzed [19].

Twelve percent of the rejected feedback only provided a quantitative rating with no narrative comment. Medicosearch requires that both a quantitative rating and a narrative comment be provided in every patient feedback, and this is likely the reason for rejecting this feedback. Narrative comments often provide a richer source of information than quantitative ratings [10]; however, making narrative comments mandatory seems inappropriate. Some patients may not be willing or able to describe what happened in a narrative comment, but may still want to share their satisfaction with their physician with others. It is unclear why these ratings should simply be excluded because the patient did not want to also write a narrative comment.

In terms of the evaluation tendencies of online patient feedback, previous Swiss and international research has found that the published online patient feedback on PRWs is overwhelmingly positive [8,10,14,17-32]. However, recent research also raised concerns that negative feedback is being suppressed by Swiss PRWs [19]. For instance, the PRW OkDoc explicitly states on its website that any negative comments will be deleted, and while Medicosearch allows negative comments, it informs the concerned physician before publishing it online, so the physician can decide whether to activate the negative feedback function [19]. There was therefore an expectation that the majority of the rejected feedback would be negative. However, this analysis of 2352 rejected feedback from Medicosearch found that just over half of all rejected feedback were negative, and the average rejected rating was 2.8 out of 5.

The proportion of rejected negative feedback, however, is substantially higher than the proportion of negative feedback that has been published in the international literature [8,10,14,17-32]. Analysis of the published feedback for a sample of Swiss physicians also reported that only 4.3% (10/234) of the feedback published on Medicosearch was negative and that the average rating was 4.68 out of 5. It is unclear why there is such a large discrepancy between published and rejected negative feedback. It has previously been suggested that Switzerland’s restrictive legal framework regarding data protection may be having a big impact on the types of online patient feedback that are published [18]. However, it may also be that PRWs like Medicosearch are also deciding themselves not to publish a lot of the negative feedback they receive owing to conflicts of interest. Medicosearch has shifted its business strategy toward online appointments, where physicians pay a fee and their booking systems are integrated with Medicosearch, which allows patients to book an appointment with a physician directly on Medicosearch. Consequently, Medicosearch will likely be reluctant to upset paying physicians by publishing too much negative feedback, as their business is now reliant on physicians using their online appointment system.

Users of PRWs can also manipulate online patient feedback, and there is some indication that physicians or practice staff sometimes pose as patients on PRWs to post either positive comments about themselves or negative comments about competitors [33]. Indeed, 25% (588/2352) of the rejected feedback were positive, and it is possible some of these were rejected because Medicosearch suspected that these were fake reviews. However, without a clear and consistent way to determine which feedback is rejected, there is a danger that feedback will be inappropriately rejected.

With regard to the contents of narrative comments, the most frequently mentioned issues identified from the rejected feedback included (1) satisfaction with treatment; (2) the overall assessment of the physician; (3) recommending the physician; (4) the physician’s communication; (5) the physician’s caring attitude; and (6) the physician’s friendliness. In comparison, the top five mentioned issues identified in the analysis of the published feedback for a sample of Swiss physicians were (1) the overall assessment of the physician and the physician’s competence; (2) the physician’s communication; (3) recommending the physician; (4) the physician’s friendliness; and (5) the physician’s caring attitude [19]. This suggests that online patient feedback is raising similar issues, regardless of whether it is published or rejected. Indeed, just like in the analysis of the published feedback for a sample of Swiss physicians [19], it is important to recognize that 95% (42/44) of the subcategories of the categorization framework and 81.5% (3101/3804) of the distinct issues identified were concerning aspects of performance (interpersonal skills of the physician and staff, infrastructure, and organization and management of the practice) that are considered to be assessable by patients.

If online patient feedback is going to be collected, there needs to be clear policies and practices about how this is handled. It cannot be left to the whims of PRWs, who may have financial incentives to suppress negative feedback, to decide which feedback is or is not published online. It has previously been recommended that “there is a need for consensus-based criteria that applies to all Swiss PRWs for determining which comments are and are not to be publicly published and which are clearly publicized so users of PRWs are aware of it” [19]. This analysis of 2352 rejected feedback from Medicosearch further highlights the need for such a consensus-based criteria. To support this, further research is needed to examine how many Swiss PRWs are using criteria for determining which feedback is published or not, what those criteria are, and what measures PRWs are using to address the manipulation of online patient feedback. It appears that research examining these issues would be helpful in most countries that have PRWs.

### Limitations

This study has some limitations that should be taken into account when interpreting the results. First, it is unknown how many patient feedback Medicosearch received in total during the time period covered. The author contacted Medicosearch asking for this information but never received a response, and the information is not freely available on the website. It would be helpful to know the proportion of patient feedback that is being rejected. Second, the sample of rejected feedback was only taken from one Swiss PRW. Although Medicosearch is one of the oldest and most used Swiss PRWs, it is unclear how generalizable the results are to other PRWs and other countries. Future research examining whether PRWs are using criteria for determining which feedback is published or not should include all Swiss PRWs. Third, the specialty and sociodemographic information of the rated physicians are unknown, and there may be important differences between the different specialties and physicians. Finally, the sociodemographic information of the rating patients is unknown and may not be representative of Swiss patients in general.

## Appendix

Multimedia Appendix 1 Categorization of issues by language.

## Abbreviations

PRW: physician rating website
