# Supplementary material for: Rejected Online Feedback From a Swiss Physician Rating Website Between 2008 and 2017: Analysis of 2352 Ratings
Source: J Med Internet Res. 2020 Aug 3;22(8):e18374. doi: 10.2196/18374 (PMC7432139; doi:10.2196/18374)
Supplement: Multimedia Appendix 1 [file jmir_v22i8e18374_app1.docx]

## **Multimedia Appendix 1. Categorisation of issues by language**

| **Issue** | **Region (%)** | **Chi-squared-test** | **Evaluation**  **% (+/=/-)** |
| --- | --- | --- | --- |
| **Physician (N=)** | | | |
| Satisfaction with treatment | German: 450/1623 (27.7)  French: 66/241 (27.4) | χ^2^_(1)_=.01,  *P*=.94 | 63(14) / 49(10.9) / 338(75.1)  16(24.2) / 9(13.6) / 41(62.1) |
| Overall assessment | German: 323/1623 (19.9)  French: 61/241 (25.3) | χ^2^_(1)_=3.8,  *P*=.06 | 101(31.3) / 52(16.1) / 170(52.6)  20(32.8) / 7(11.5) / 34(55.7) |
| Recommendation | German: 299/1623 (18.4)  French: 44/241 (18.3) | χ^2^_(1)_=.004,  *P*=1.0 | 32(10.7) / 37(12.4) / 230(76.9)  3(6.8) / 9(20.5) / 32(72.7) |
| Communication | German: 212/1623 (13.1)  French: 42/241 (17.4) | χ^2^_(1)_=3.4,  *P*=.07 | 29(13.7) / 15 (7.1) / 168(79.2)  8(19) / 4(9.5) / 30(71.4) |
| Caring attitude | German: 186/1623 (11.5)  French: 29/241 (12) | χ^2^_(1)_=07,  *P*=.83 | 32(17.2) / 11(5.9) / 143(76.9)  7(24.1) / 1(3.4) / 21(72.4) |
| Friendliness | German: 186/1623 (11.5)  French: 15/241 (6.2) | **χ^2^_(1)_=5.9,**  ***P*=.01** | 33(17.7) / 15(8.1) / 138(74.2)  2(13.3) / 2(13.3) / 11(73.3) |
| Treatment cost/billing | German: 154/1623 (9.5)  French: 18/241 (7.5) | χ^2^_(1)_=1.0,  *P*=.34 | 7(4.5) / 24(15.6) / 123(79.9)  1(5.6) / 6(33.3) / 11(61.1) |
| Competence | German: 147/1623 (9.1)  French: 20/241 (8.3) | χ^2^_(1)_=.15,  *P*=.81 | 36(24.5) / 12(8.2) / 99 (67.3)  7(35) / 1(5) / 12(60) |
| Being taken seriously | German: 133/1623 (8.2)  French: 7/241 (2.9) | **χ^2^_(1)_=8.5,**  ***P*=.002** | 10(7.5) / 8(6.0) / 115(86.5)  0 / 2(28.6) / 5(71.4) |
| Time spent with patient | German: 118/1623 (7.3)  French: 15/241 (6.2) | χ^2^_(1)_=.35,  *P*=.69 | 15(12.7) / 17(14.4) / 86(72.9)  2(13.3) / 1(6.7) / 12(80) |
| Trust | German: 109/1623 (6.7)  French: 21/241 (8.7) | χ^2^_(1)_=1.3,  *P*=.27 | 32(29.4) / 7(6.4) / 70(64.2)  11(52.4) / 3(14.3) / 7(33.3) |
| Professionalism | German: 83/1623 (5.1)  French: 13/241 (5.4) | χ^2^_(1)_=.03,  *P*=.88 | 4(4.8) / 6(7.2) / 73(88)  7(53.8) / 0 / 6(46.2) |
| Cooperation with medical specialists | German: 11/1623 (0.7)  French: 2/241 (0.8) | χ^2^_(1)_=.07,  *P*=.68 | 3(27.3) / 0 / 8(72.7)  1(50) / 0 / 1(50) |
| Alternative medicine | German: 7/1623 (0.4)  French: 1/241 (0.4) | χ^2^_(1)_=.001,  *P*=1.0 | 5(71.4) / 0 / 2(28.6)  1(100) / 0 / 0 |
| Telephone availability | German: 6/1623 (0.4)  French: 2/241 (0.8) | χ^2^_(1)_=1.0,  *P*=.27 | 1(16.7) / 2(33.3) / 3(50)  0 / 1(50) / 1(50) |
| Privacy | German: 7/1623 (0.4)  French: 0/241 | χ^2^_(1)_=1.0,  *P*=.61 | 0 / 2(28.6) / 5(71.4)  0 / 0 / 0 |
| Health insurance differentiation | German: 3/1623 (0.2)  French: 3/241 (1.2) | **χ^2^_(1)_=7.3,**  ***P*=.03** | 0 / 0 / 3(100)  0 / 0 / 3(100) |
| Patient involvement | German: 5/1623 (0.3)  French: 0/241 | χ^2^_(1)_=7.4,  *P*=1.0 | 0 / 1(20) / 4(80)  0 / 0 / 0 |
| Individualized service | German: 2/1623 (0.1)  French: 0/241 | χ^2^_(1)_=.29,  *P*=1.0 | 0 / 0 / 2(100)  0 / 0 / 0 |
| Child friendliness | German: 1/1623 (0.1)  French: 0/241 | χ^2^_(1)_=.15,  *P*=1.0 | 0 / 0 / 1(100)  0 / 0 / 0 |
| **Staff (N=)** | | | |
| Friendliness | German: 103/1623 (6.3)  French: 5/241 (2.1) | **χ^2^_(1)_=7.0,**  ***P*=.005** | 38(36.9) / 21(20.4) / 44(42.7)  0 / 2(40) / 3(60) |
| Overall assessment | German: 48/1623 (3)  French: 12/241 (5) | χ^2^_(1)_=2.8,  *P*=.12 | 15(31.3) / 11(22.9) / 22(45.8)  4(33.3) / 7(58.3) / 1(8.3) |
| Service/assistance | German: 30/1623 (1.8)  French: 1/241(0.4) | χ^2^_(1)_=2.6,  *P*=.17 | 10(33.3) / 3(10) / 17(56.7)  1(100) / 0 /0 |
| Communication | German: 20/1623 (1.2)  French: 2/241 (0.8) | χ^2^_(1)_=.29,  *P*=1.0 | 4(20) / 5(25) / 11(55)  0 / 2(100) / 0 |
| Professionalism | German: 9/1623 (0.6)  French: 1/241(0.4) | χ^2^_(1)_=.07,  *P*=1.0 | 2(22.2) / 2(22.2) / 5(55.6)  0 / 0 / 1(100) |
| Availability by telephone | German: 5/1623 (0.3)  French: 1/241 (0.4) | χ^2^_(1)_=.08,  *P*=.57 | 1(20) / 3(60) / 1(20)  0 / 1(100) / 0 |
| Time spent with patient | German: 2/1623 (0.1)  French: 0/241 | χ^2^_(1)_=.30,  *P*=1.0 | 1(50) / 0 / 1(50)  0 / 0 / 0 |
| Health insurance differentiation | German: 0/1623  French: 1/241 (0.4) | χ^2^_(1)_=6.7,  *P*=.13 | 0 / 0 / 0  0 / 1(100) / 0 |
| Trust | German: 1/1623 (0.1)  French: 0/241 | χ^2^_(1)_=.15,  *P*=1.0 | 0 / 0 / 1(100)  0 / 0 / 0 |
| **Practice (N=)** | | | |
| Overall assessment | German: 251/1623 (15.5)  French: 38/241 (15.8) | χ^2^_(1)_=.02,  *P*=.92 | 97(38.6) / 76(30.3) / 78(31.1)  14(36.8) / 12(31.6) / 12(31.6) |
| Waiting time within practice | German: 145/1623 (8.9)  French: 10/241 (4.1) | **χ^2^_(1)_=6.3,**  ***P*=.01** | 18(12.4) / 39(26.9) / 88(60.7)  4(40) / 3(30) / 3(30) |
| Atmosphere | German: 90/1623 (5.5)  French: 4/241 (1.7) | **χ^2^_(1)_=6.6,**  ***P*=.007** | 37(41.1) / 27(30) / 36(28.9)  1(25) / 2(50) / 1(25) |
| Organization | German: 34/1623 (2.1)  French: 3/241 (1.2) | χ^2^_(1)_=.78,  *P*=.62 | 6(17.6) / 11(32.4) / 17(50)  1(33.3) / 0 / 2(66.7) |
| Ability to get appointment | German: 33/1623 (2)  French: 2/241 (0.8) | χ^2^_(1)_=1.6,  *P*=.31 | 4(12.1) / 10(30.3) / 19(57.6)  0 / 0 / 2(100) |
| Equipment | German: 25/1623 (1.5)  French: 6/241 (2.5) | χ^2^_(1)_=1.2,  *P*=.28 | 6(24) / 6(24) / 13(52)  2(33.3) / 4(66.7) / 0 |
| Recommendation | German: 18/1623 (1.1)  French: 6/241 (2.5) | χ^2^_(1)_=3.1,  *P*=.11 | 4(22.2) / 3(16.7) / 11(61.1)  0 / 2(33.3) 4(66.7) |
| Consultation hours | German: 7/1623 (0.4)  French: 1/241 (0.4) | χ^2^_(1)_=.001,  *P*=1.0 | 2(28.6) / 3(42.9) / 2(28.6)  1(100) / 0 / 0 |
| Location | German: 7/1623 (0.4)  French: 0/241 | χ^2^_(1)_=1.0,  *P*=.61 | 2(28.6) / 2(0.7) / 3(0.3)  0 / 0 / 0 |
| Waiting room entertainment | German: 5/1623 (0.3)  French: 0/241 | χ^2^_(1)_=.74,  *P*=1.0 | 4(80) / 1(20) / 0  0 / 0 / 0 |
| Parking space | German: 3/1623 (0.2)  French: 0/241 | χ^2^_(1)_=.45,  *P*=1.0 | 3(100) / 0 / 0  0 / 0 / 0 |
| Availability by telephone | German: 3/1623 (0.2)  French: 0/241 | χ^2^_(1)_=.45,  *P*=1.0 | 1(33.3) / 1(33.3) / 1(33.3)  0 / 0 / 0 |
| Privacy | German: 2/1623 (0.1)  French: 1/241 (0.4) | χ^2^_(1)_=1.1,  *P*=.34 | 0 / 2(100) / 0  0 / 1(100) / 0 |
| Barrier free access | German: 2/1623 (0.1)  French: 0/241 | χ^2^_(1)_=.29,  *P*=1.0 | 0 / 1(50) / 1(50)  0 / 0 / 0 |
| Online appointment | German: 1/1623 (0.1)  French: 0/241 | χ^2^_(1)_=.15,  *P*=1.0 | 0 / 1(100) / 0  0 / 0 / 0 |
